# Domestic Summary

**Published:** 1839-09-14

**Authors:** 


### DOMESTIC SUMMARY.

*American Authors in England.*—Dr. Meigs' Philadelphia Practice of Midwifery, and Dr. Harlan's Medical and Physical Transactions, have been favourably reviewed, the first in the British and Foreign Medical Review, the second in Dr. J. Johnson's Medico-Chirurgical Review, for July of this year.

*Cincinnati Medical College.*—We noticed lately the resignation of Professor Gross. The remaining professors have since resigned, and the medical department of the College is for the present suspended.

*Professor Griffith.*—We learn that the University of Virginia is about to lose the valuable services of Professor Griffith, who intends retiring in consequence of ill health.

*The Albany Medical College and the Thompsonians.*—A notice appeared, some time since, in the American Medical Intelligencer, copied into the American Journal of the Medical Sciences, calling attention to a presumed coalition between the Albany Medical College and the Thompsonians. The following explanation of the affair, from the President of the College, appears to be satisfactory:—

"The Thompsonians, during their meeting in Albany, requested permission to visit the Albany Medical College, which was granted to them as to other persons who apply for the same favour. While there, they expressed to Dr. March their intention to recommend to their students to acquire a more thorough knowledge of 'anatomy, physiology, surgery and chemistry,' and asked on what terms they would be received into the institution. Dr. March replied, that they would be received on the same terms as any other persons. It was neither intended by Dr. M., nor supposed by those who made the inquiry, that the Thompsonian students would be admitted to graduate, or be allowed any privileges which they would not enjoy in any other medical institution. For we suppose that no institution would refuse to admit an applicant to attend the lectures, simply because he might be a student of a Thompsonian doctor.

"The charter of the Albany Medical College expressly enjoins, among other requisites for graduation, 'that the student shall have pursued the study of medical science for at least three years after the age of sixteen, with some physician and surgeon duly authorized by law to practise the profession;' so that it would be out of the power of the faculty and trustees to grant degrees to Thompsonian students, even if they were disposed to form an alliance with them, such as, from Dr. Dunglison's remarks, he seems to suppose exists. Any other privilege but that of graduation, they would enjoy in common with other students in the Albany Medical College, as in other medical colleges in this country."—*Boston Journal.*

